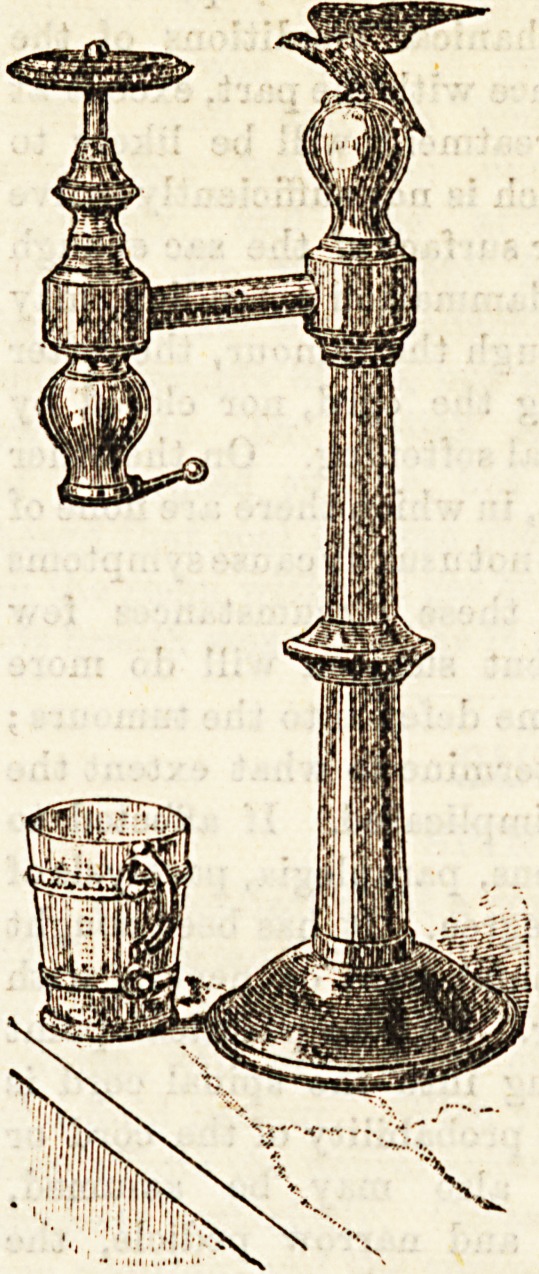# New Drugs, Appliances, and Things Medical

**Published:** 1890-07-26

**Authors:** 


### NEW DRUGS, APPLIANCES, AND THINGS MEDICAL.

[All preparations, appliances, novelties, etc., of which a notice is desired, should be sent to The Editor, The Lodge, Porchester Square, W.]

### AERATED WATERS, &c.

In continuance of our inspection of the different exhibits at the Royal Military Exhibition we now notice the very complete and interesting one of Messrs. Barnett and Foster. This has been rendered all the more easy by the care and attention in explanation given by the assistant at the stand. We first come to the carbonic acid gas generator. In this machine the basis of all effervescing drinks is made. The manufacturers claim for it that it is the most economical apparatus in the market, and that by its use 50 per cent. in materials and wages can be saved. Amongst its other advantages may be mentioned the following : (*a*) The atmospheric air is excluded. (*b*) The gas is washed, purified, and cooled ; thus the waters produced by its use are of better quality. (*c*) Great saving of time, as when once supplied it is entirely automatic in its action. (*d*) Great compactness, containing nothing to wear out, only one mixing rod to go from top to bottom. (*e*) So simple in its construction, that it cannot get out of order.

Having been shown the generator, we next had the soda-water machine explained to us. This "Niagara" machine dispenses with the mechanical stirrer or agitator, simplifying the process in its mechanical arrangement, thus preventing the liability to derangement in the working of the machine. By reference to the section of which we give an engraving, it will be seen that the gas and water are most thoroughly mixed at a high pressure. They are both forced up by the pump in the space B, to fall through the perforated plate C into the inner division E of the cylinder, this division being also surcharged with the gas ; the water in its state of fine division has thus every chance of being fully charged with the gas.

The firm supply cylinders full of the liquefied gas, so that small manufacturers can make their own aërated waters without the trouble and expense of making their own gas.

Again, these cylinders of compressed and liquefied gas lend themselves greatly to the furtherance and development of businesses where iced fruit and other effervescing drinks are dispensed. To chemists and others who have not their own soda water machines, nothing can be more convenient for supplying the reservoir for counter fountains than these cylinders of compressed gas. It enables the dispenser to have the control in his own hands instead of having to send away the cylinder to be charged with aërated water. The only expense is the cost of the vessel of liquefied gas. The agent informed us that the firm sends out these "bottles of liquefied gas" to any part of the United Kingdom at very reasonable rates. Another arrangement for using the liquefied gas for chemists and others doing a syphon or bottle retail trade is shown by what the firm calls a "Compactum" machine. In the advertisement of the firm appearing in this issue we show an illustration of it. By this machine 700 to 1,200 bottles can be filled in an hour. The space required is only 8 feet long by 3 feet wide. The gas supplies itself automatically, so that, beyond the bottler, all the work to be done is represented in the pumping of the water into the proper receptacle. We were next taken to the counter fountains. The simplest of these we show. By it a small, sharp stream of aërated water can be drawn, which, when syrups are used, is absolutely necessary in order to produce a properly made mixture of them and the water, afterwards a larger stream can be produced, as in the emptying of a bottle of soda water. Where soda water alone is required, by the use of this apparatus all the sharpness and pungency is retained and none of the gas lost, as is done in first filling a bottle and then emptying it. At Chelsea we were shown a very elaborate silver-plated apparatus which seemed to be well patronized. This had eight varieties of syrup, an ice-shaver, besides the soda fountain. We might describe a great deal more we saw, but our space forbids us, more especially so when we find Messrs. Barnett and Foster's catalogue contains no less than 200 pages. We were much pleased with the exhibit, and strongly recommend hospital authorities, mess committees, and all those who use large quantities of aërated waters, to inspect it thoroughly. They will receive every attention from Colonel Foster or his indefatigable assistant. We may mention, in conclusion, as a decided evidence of well-earned success that no less than 250 complete sets of apparatus have been sold to the messes of different regiments.

## Figures and Tables

**Figure f1:**
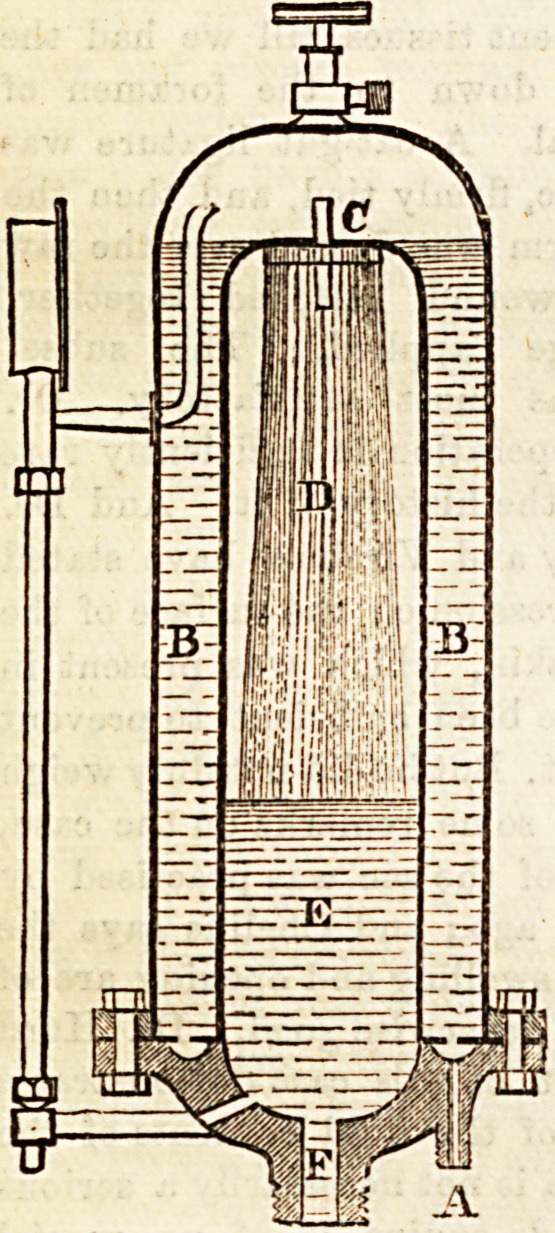


**Figure f2:**